# Towards Highly Efficient TADF Yellow-Red OLEDs Fabricated by Solution Deposition Methods: Critical Influence of the Active Layer Morphology

**DOI:** 10.3390/nano10010101

**Published:** 2020-01-04

**Authors:** Manish Kumar, Luiz Pereira

**Affiliations:** 1 Department of Physics and i3N—Institute for Nanostructures, Nanomodulation and Nanofabrication, University of Aveiro, 3810-193 Aveiro, Portugal; mkumar@ua.pt; 2 CeNTI—Centre for Nanotechnologies and Smart Materials, R. Fernando Mesquita, 2785, 4760-034 Vila Nova de Famalicão, Portugal

**Keywords:** solution-processed OLEDs, TADF, morphology, electrical transport improvement, efficiency

## Abstract

Organic light-emitting diodes (OLEDs) based on thermally activated delayed fluorescence emitters (TADF) in simple device structures fabricated by solution processing are strongly dependent on a suitable host molecular conformation and morphology. Herein, we report the fabrication of highly efficient yellow-red TADF-based OLEDs via solution processing in a simple, two-organic-layer device structure. The devices were fabricated at different weight concentrations of 5%, 8%, and 10% emitter in an n-/p-type mixed host matrix, and their characteristics were studied. The device performance was compared with different thickness parameters for both the emitting layer (EML) and the electron transport layer (ETL) in various solvents, including chlorobenzene, dichlorobenzene, and chloroform. By optimizing the mixed ratio of EML, yellow-red OLEDs of 2-[4 (diphenylamino)phenyl]-10,10-dioxide-9*H*-thioxanthen-9-one (TXO-TPA) emitter in an n-/p-type host matrix of poly(*N*-vinylcarbazole):1,3-Bis[2-(4-tert-butylphenyl)-1,3,4-oxadiazo-5-yl]benzene (PVK:OXD-7) as a blend for the active layer were fabricated. In the best results, the device exhibited a lower turn-on voltage at around 6 V, with an external quantum efficiency (EQE) of 18.44%, current efficiency of 36.71 cd/A, and power efficiency of 14.74 Lm/W for the 8% emitter concentration. The importance of solvent for improving the electrical properties, together with organic layer thickness and host effect for the charge carrier’s transport and device characteristics are also discussed.

## 1. Introduction

Organic light-emitting diodes (OLEDs) have emerged as an extensive active research topic in terms of both their scientific and technological aspects, and the improvement of device efficiency, stability, and cheapness and simple device fabrication processes have been a topic of interest. They have attracted considerable attention due to their promising applications in cheap, energy-saving, eco-friendly and solid-state lighting [1,2,3,4].

For the fabrication of OLEDs, both solution-processing and thermal evaporation methods have been used, and the choice of fabrication process has driven OLED research aiming to maximize the use of materials. Solution-process fabrication of OLEDs is an exceptionally rapidly evolving technology with applications in both the display and lighting industries [4,5]. The thermal vacuum evaporation process grants excellent stability and exceptional control of organic layers, but always involves a more complex structure to enable efficient charge carrier transport and recombination. As a drawback, this process involves high vacuum generation requirements and complex procedures, which results in high costs for fabrication of large areas [5,6,7]. In spite of this, a good electrically balanced device can be obtained, but sometimes more than five layers need to be used. Therefore, for practical applications, a drastic reduction of device structure complexity is required, even if a collateral loss of efficiency is associated. The final results can still demonstrate high performance. Thus, solution-process methods, such as spin-coating, inkjet process, screen-printing, and roll-to-roll methods, are enticing for low-cost large-area fabrication [8,9], as the modulation of electrical behavior in active layer becomes far easier, although still not straightforward.

The emergence of thermally activated delayed fluorescence (TADF) organic emitters has generated considerable attention regarding their application in organic light-emitting diodes. In conventional organic emitters, the internal quantum efficiency is limited to 25% because only a quartet of singlet (*S*) excitons can recombine radiatively, and the remaining triplet (*T*) excitons, which undergo non-radiative relaxation, cannot generate photons. In this context, and to respond to this need, the chemical development of organic TADF materials, first reported by Adachi et al. in 2012 [10], appears to be a suitable solution. In TADFs, the pure organic aromatic system exhibits a very low energy gap (*ΔE_ST_*) between the *S* and *T* levels, enabling the up-conversion of triplet excitons to singlet excitons, where the Internal Quantum Efficiency (IQE) can easily reach up to 100% [11,12]. These values of IQE are similar to those found in more expensive and complex transition metal compounds generated via spin-orbit coupling (SOC) [13]. Currently, TADF emitters are considered to be the third generation of OLEDs. Nevertheless, the optimization of TADF-based OLEDs is not straightforward, and great efforts have been made to enhance the performance of these devices via molecular design strategies and device structure modulation [11,12,14].

One of the main goals with TADF-based OLEDs is to take advantage of high IQE of the emitter, and simultaneously to decrease the device complexity while retaining high external efficiencies. Moreover, these materials must follow the concept of host:guest active layer architecture. Again, this is not simple, although the manipulation of the host materials currently appears to be a very suitable and reliable process [15]. The most promising possibility is to employ solution-deposition methods, where the electrical carrier properties can be easily manipulated. However, this requires substantial effort to guarantee correct host:guest molecular ordering and produce the desired results.

Nevertheless, the concept of solution-deposited OLEDs based on highly efficient emitters is not new. For instance, a solution-processed phosphorescent tandem OLED exhibiting extremely small efficiency roll-off was recently reported with 22% external quantum efficiency (EQE), but the structural complexity was too high for large-area applications [16]. In the present state of the art, the efficiency of thermally processed devices has already reached 36.7% and 37.8% for blue and green emitters, respectively [17,18,19]. In solution-processed devices, an EQE of 31% has been reported with the use of a cross-linkable polymer in blue-green TADFs, but the process again involves a complex processing method [20]. Recently, reports on the solution-processed and thermally evaporated TADF-based OLEDs have summarized recent advances in such processes, but they have failed to deliver effective charge transport properties and recombination processes inside the emissive layer [15,21,22,23,24]. Typically, research in this area is still in an early stage, as there is a lack of standardization for the fabrication and performance evaluation of these devices. First, the use of TADFs still presents a challenge in the charge-transfer process at the interfaces with further consequence in the ETL and EML thickness [18,25,26,27]. Second, the choice of host materials and respective HOMO/LUMO (highest occupied molecular orbitals, and lowest unoccupied molecular orbitals) and triplet energy levels is not simple and, in some cases, can results in high hole-injection barrier formation, resonant non-radiative loss, and bad film formation that degrades the electrical carrier transport and confinement in the active layer [28], as well as drastically changing the electrical carrier’s mobility. Third, triplet–triplet annihilation due to long triplet decay lifetime in the EML is still a real problem [18,29].

Some of these issues need further investigation. In solution-processed organic devices, the solvent plays a critical role in the device performance, where the viscosity, temperature, and evaporation rate of solvent control the electrical properties of the thin film [30,31,32,33,34,35]. A non-aromatic solvent such as chloroform (CF), with a high evaporation rate, or an aromatic solvent like chlorobenzene (CB) or dichlorobenzene (DCB), with a low evaporation rate, can be employed to study device characteristics, and exploiting these properties could be an effective route to change the electrical and optical properties of the device [34].

In this work, we demonstrated the effect of solvents on device performance for a stable, highly efficient TADF-based OLED. The solvents (chloroform, dichlorobenzene, and chlorobenzene) were chosen according to their distinctly different evaporation rates (allowing the development of different organic film morphology and molecular stacking order) and, because all materials used had high solubility, avoiding bad film formation by spin-coating. We first investigated the effect of the weight concentration of small-molecule-based yellow-red emitter 2-[4 (diphenylamino)phenyl]-10,10-dioxide-9H-thioxanthen-9-one (TXO-TPA) in an n-/p-type host matrix of poly(N-vinylcarbazole):1,3-Bis[2-(4-tert-butylphenyl)-1,3,4-oxadiazo-5-yl]benzene (PVK:OXD-7) as a blend for the active layer. This emitter was reported by Wang et al. in 2014 [25] in a three-organic-layer structure with a maximum EQE of 18.5% in a thermally deposited device. We chose this red emitter because it has already been demonstrated to harvest nearly 100% of the excited triplets via TADF with a quantum yield (PLQY) of 83% in a coevaporated film with 5 wt.% TXO-TPA:mCP (1,3-Bis(N-carbazolyl)benzene) film. However, no investigation of solution-processed devices using this emitter has been done. Moreover, the complexity of the thermally deposited device was too high. Additionally, we demonstrated the electrical transport and confinement optimization of the emissive layer when different solvents were used. Different guest TXO-TPA concentrations 5, 8, and 10 wt.% were studied to identify the most efficient emission. The relationship of wt.% of emitter and thickness of organic layers with current density and electrical transport carrier was investigated in detail. The device incorporating 8 wt.% TXO-TPA showed a maximum current efficiency (η_c_) of 36.71 cd/A, a maximum power efficiency (η_p_) of 14.18 Lm/W, and an EQE of 18.44%. The red-light-emitting devices were compared with their reported vacuum-evaporated counterparts. Additionally, the photophysical characteristics of the blends at different wt.% were investigated in chlorobenzene, PVK:OXD-7, and Zeonex matrices to find the relationship between the emitter concentration, thickness, and photoluminescence quantum yield (PLQY). An attempt has been to explain the results, considering the influence of the different solvents in the electrical carrier transport and recombination. To the best of our knowledge, no solution-processed OLEDs have been previously reported using this red emitter in a simple device structure.

## 2. Materials and Methods

### 2.1. Materials

The TADF emitter (TXO-TPA (2-[4-(diphenylamino)phenyl]-10,10-dioxide-9*H*-thioxanthen-9-one)), p-type polymer PVK (poly(*N*-vinylcarbazole), and n-type molecule OXD-7 (1,3-Bis[2-(4-tert-butylphenyl)-1,3,4-oxadiazo-5-yl]benzene) were obtained from Lumtech Ltd., New Taipei, Taiwan. The n-type molecules TmPyPb (1,3,5-Tri(m-pyridin-3-ylphenyl)benzene) and TPBi (2,2′,2′′-(1,3,5-Benzinetriyl)-tris(1-phenyl-1-*H*-benzimidazole), hole-injection polymer PEDOT:PSS (poly(styrenesulfonate)-doped poly(3,4-ethylenedioxythiophene), and LiF (lithium fluoride) were obtained from Ossila Ltd., Sheffield, UK. All solvents were bought from Sigma Aldrich, Darmstadt, Germany. The patterned Indium Tin Oxide (ITO) substrates (sheet resistance of 20 Ω/□) were bought from Ossila Ltd. UK. All organic materials were sublimed before use.

### 2.2. Device Fabrication

**Figure 1:**
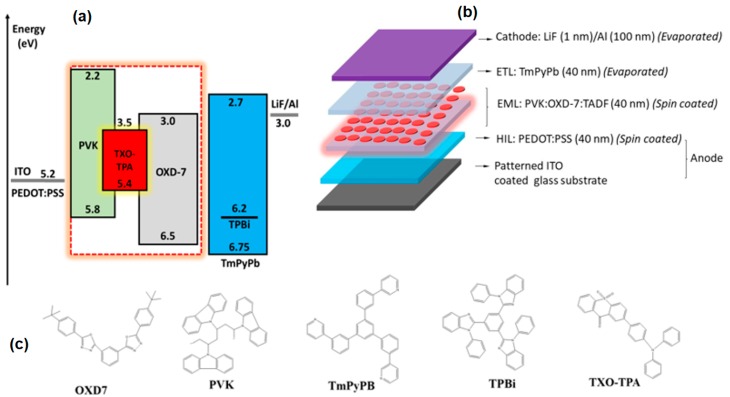
(**a**) The energy diagram and (**b**) the device structure used in this work; (**c**) the molecular structures of 1,3-Bis[2-(4-tert-butylphenyl)-1,3,4-oxadiazo-5-yl]benzene) (OXD-7), poly(N-vinylcarbazole (PVK), 1,3,5-Tri(m-pyridin-3-ylphenyl)benzene TmPyPb, 2,2′,2′′-(1,3,5-Benzinetriyl)-tris(1-phenyl-1-*H*-benzimidazole (TPBi), and 2-[4-(diphenylamino)phenyl]-10,10-dioxide-9*H*-thioxanthen-9-one (TXO-TPA).

The simple device structure was made of glass/ITO/PEDOT:PSS (40 nm)/EML (x nm)/ETL (y nm)/LiF (1 nm)/Al (100 nm). The emissive layer, was [PVK:OXD-7]_(100-a)_:TXO-TPA_a_, where *a* was the wt.% of the TADF-emitting dopant in the host PVK:OXD-7 (3:2 wt.%, respectively). The substrates were sequentially cleaned in an ultrasonic bath containing Hellmanex solution, acetone, and 2-propanol (IPA), and with a UV ozone treatment for 5 min before use. Prior to deposition, the PEDOT: PSS was filtered with a 0.45 μm polyvinylidene difluoride (PVDF) filter. The PEDOT: PSS layer (hole-injection layer—HIL) was spin-casted dynamically at 4200 rpm and annealed at 120 °C for 15 min. The EML [PVK:OXD-7]_(100-a)_:TXO-TPA_a_ layer (CB, DCB, and CF solvent) was spin-coated at a different rpm after being filtered using 0.1 μm polytetrafluoroethylene (PTFE) filter and dried in the glove box at 80 °C for 30 min. The spin-coating parameters were adjusted in order to achieve the final desired organic layer thickness, which was measured by profilometry. The solution concentration (total) was 42 mg/mL. An electron transport layer of TmPyPb or TPBi was then thermally deposited on the EML, along with the dielectric/metal layers of 1 nm LiF and 100 nm Al, in a vacuum, at a pressure lower than 5 × 10^−6^ mBar. The rates for organic material deposition was 1 Å/s, 0.1 Å/s for LiF and 2Å/s for Al. The device active area was 4.5 mm^2^. This structure, regardless of PEDOT:PSS (metal-like polymer) represented a two-organic-layer device only. Figure 1 shows the general device scheme and the energy level diagram.

### 2.3. Optical and Electrical Measurements

The current–voltage–luminance (J-V-L) characteristics were determined using a Keithley Source-Meter 2425 model (Tektronix Inc., Beaverton, OR, USA) and a Minolta LS-100 Luminance Meter (Konica Minolta Inc., Tokyo, Japan). For the electroluminescence spectra, an Ocean Optics USB4000 spectrometer (Ocean Optics Inc., Largo, FL, USA) was used with the sensitivity response set in the wavelength range 350–950 nm. Power and current efficiency and EQE were calculated assuming a Lambertian emission profile. Photoluminescence spectra of studied samples were gathered at room temperature with an Edinburgh Instruments FLS980 fluorescence spectrometer (Edinburgh Instruments Ltd., Livingstone, UK) with a Xe lamp as an excitation source and an R-928 photomultiplier detector. The PLQY values of compounds were measured using an integrating sphere from Edinburgh Instruments with a BENFLEC coating. All the measurements and device characterizations (without any encapsulation) were made in the ambient atmosphere at room temperature.

## 3. Results and Discussion

### 3.1. Photophysical Characterization and Film Morphology

**Figure 2:**
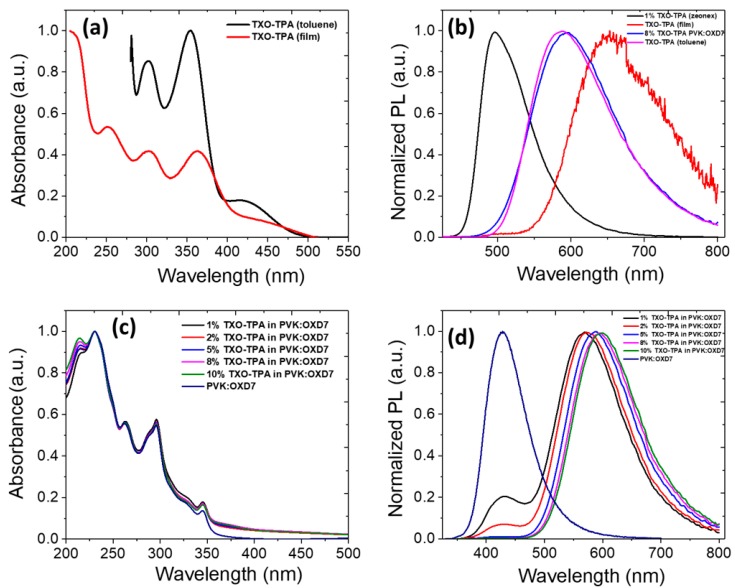
(**a**) Normalized absorption spectra of the TXO-TPA in toluene solution (10^−5^ M) and in a pristine film; (**b**) photoluminescence spectra of TXO-TPA in Zeonex, pristine film, 8 wt.% TXO-TPA in PVK:OXD-7, and in toluene solution. (**c**) Normalized absorption spectra of thin films of PVK:OXD-7 doped with different amounts of TXO-TPA and pure PVK:OXD-7 host, and (**d**) normalized photoluminescence spectra of thin layers: the neat PVK:OXD-7 matrix and PVK:OXD-7 doped with 1, 2, 5, 8, 10 wt.% emitter molecules.

For TXO-TPA, the UV–vis absorption photoluminescence spectra are shown in Figure 2. Figure 2a shows the absorption spectra of TXO-TPA in its solid state and in toluene solution. The absorption spectra of the D–A molecule showed a major peak at 420 nm, which was assigned to charge transfer absorption (CT) mainly associated with electron transport from the TPA moiety to the TXO moiety, and additional peaks at 350 nm, 300 nm, and 250 nm clearly reflected the sum of the TXO and TPA moieties [25]. The compound exhibited emissive spectra at 590 nm in toluene, which were red-shifted to 650 nm in the thin film due to aggregation-induced emission. The absolute fluorescence quantum yield (Φ_PL_) was 40%.

However, in the nonpolar rigid media Zeonex, this red emitter showed a blue-shifted emission at 500 nm, which could be explained by an excited state with a strong ^1^π–π* character. Such rigid chromic effect suggests a strong intramolecular charge transfer character ^1^CT of the emissive state (Figure 2b) [36].

In such a device, the host should ensure efficient hole and electron transfer for effective charge carrier balance. The photoluminescence spectra of TXO-TPA doped into the host matrix (PVK:OXD-7) with 1, 2, 5, 8, and 10 wt.% are shown in Figure 2d, where PVK:OXD-7 was the host material.

PVK and OXD-7 will provide efficient Dexter and Foster energy transfer to the TADF dopant due to relatively high triplet and singlet levels. Some previous work has shown that this matrix can be particularly suitable for phosphorescence emitters [37,38]. Moreover, and from a practical, technological point of view, the PVK:OXD-7 blend allows a good film formation and the usual viscosity of the solution allows it to be adapted for inkjet printing or roll-to-roll process [39]. In the PVK:OXD-7 host, the D–A emission spectra demonstrated a broad Gaussian-shaped emission with the maximum 570–600 nm significantly blue-shifted at different wt.% compared to the pure film (Figure 2d). This could have been due to the rigidification of the molecular structure of TXO-TPA in the host matrix, which can lower the reorganization energy in the aggregated state [40]. It could also have been due to the weakened interactions between TXO-TPA and PVK:OXD-7 host matrix and the polarity difference of host materials [41]. The PLQY also increased up to 60% higher than the pure film (35%), indicating that the effective upconversion of triplets did occur. The value of PLQY of TXO-TPA in the pure film was 35%, and in Zeonex matrix was 100%.

Films doped with 1 and 2 wt.% TXO-TPA exhibited peak emissions of 570 and 575 nm, which correspond to the CT state. An additional peak at 425 nm was also observed, which was attributed to the emission of PVK:OXD-7. This indicates that at lower doping of TXO-TPA, we did not assure full energy transfer from the host to TXO-TPA, and the emission was the sum of both a weak host emission and the guest. At higher doping concentrations, only the emission corresponding to TXO-TPA was observed, indicating the complete transfer of the energy from PVK:OXD-7 to TXO-TPA. The PVK:OXD-7:TXO-TPA film gave a yellow emission with a peak emission wavelength of 570–590 nm, significantly blue-shifted compared with its counterpart in the pure film. The long intermolecular distance between the polar carbazole core units of PVK and the TXO-TPA weakened the solid-state solvatochromic effect, resulting in a blue-shifted emission color [41]. Upon doping concentration increase, the PLQY also increased and was measured at 60% with 8 wt.% doping. These results are typical of TADF emitters. The main principle of TADF emission lies in the high probability of reverse intersystem crossing (*rISC*) between excited *T* and *S* states due to the lower exchange energy value [10]. This gives rise to a sum of a prompt fluorescence (*PF*) with an ultra-fast decay from *S*_1_ → *S*_0_ states, and a delayed fluorescence (*DF*) with a higher lifetime, resulting from the (*T*_1_ → *S*_1_, *rISC*) → *S*_0_ transition [10,22]. Low values of PLQY in the non-degassed environment are the usual consequence of *DF* extinction in TADF emitters when in the presence of oxygen [22]. Increasing the TADF concentration in the host should give rise to an increase of the PLQY, but further decreasing is expected when the spatial density of emitters becomes high enough for quenching. In our case, using PVK:OXD-7 as the host, the optimal TXO-TPA concentration was found to be 8 wt.%. When the doping concentration increased from 1–10 wt.%, a red-shift in emission spectrum was observed, indicating the stronger direct intermolecular interaction which became important at higher concentrations. The emission of pristine PVK:OXD-7 film confirmed this observation [42,43]. The results are summarized in Table 1.

**Figure 3:**
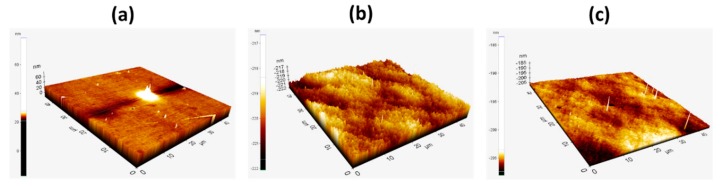
Atomic force microscopy (AFM) image of PVK:OXD-7:TXO-TPA of 8 wt.% in (**a**) chloroform, (**b**) dichlorobenzene, and (**c**) chlorobenzene.

To compare the surface morphology and effect of the solvent on thin-film formation, spin-coated thin films of PVK:OXD-7 with 5, 8, and 10 wt.% TXO-TPA were prepared in CB, DCB, and CF of 40 nm thickness on an ITO substrate. The importance of such morphology is the relationship with the device’s optic–electrical characteristics. Figure 3 shows the atomic force microscopy (AFM) images of films for 8 wt.% in CF, DCB, and CB. The root-mean-square (RMS) roughness was 1.30 nm, 0.60 nm, and 0.30 nm, respectively. As generically observed, for the same solvent, the root-mean-square roughness tended to slightly increase when the TXO-TPA concentration decreased and vice-versa. When the TADF concentration ranged from 5 to 10 wt.%, the RMS value was 0.40 nm. The uniformity of films obtained using CB as a solvent was clearly high. The differences in morphology when the TADF concentration changed in the same solvent was not noticeable (as for the case of CB and DCB, see Figure S1 in the Supplementary Materials).

### 3.2. Device Characterization

Two different but simultaneous approaches were used to optimize the device. Different solvents were used for deposition of the active layer and, for the best results, an attempt to use a different electron transport layer (ETL) was made. The different ETLs, TmPYPb and TPBi, were employed with different layer thickness as well as different EML layer thickness, and their characteristics were studied. TmPyPb [44] was primarily used as ETL due to its high electron mobility of 10^−3^ cm^2^ V^−1^ s^−1^ and high triplet E_T_ = 2.8 eV— higher than TPBi (E_T_ of 2.7 eV), which has an electron mobility of 3.3–8 × 10^−5^ cm^2^ V^−1^ s^−1^ [45]. OXD-7 has a triplet level E_T_ = 2.7 eV and electron mobility of 1–4 × 10^−5^ cm^2^ V^−1^ s^−1^ [39], whereas PVK has been used as a host material for various metal–phosphorescent complexes due to its high triplet (T_1_; 3.0 eV) energies [46,47]. However, its low hole and electron mobilities, 10^–6^ and 10^–9^ cm^2^ V^−1^ s^−1^, respectively [48], and high rate of TTA that limits the EQE, implies that pure PVK is not the best host material [49]. Thus, a blended system of PVK:OXD-7 was chosen for the EML. Besides these triplet levels, the HOMO/LUMO levels and the electrical carrier mobility in the active layer needed to be estimated for the individual materials. As previously assumed [50], it is consistent to consider that the HOMO level of the active layer should correspond to the HOMO level of the p-type material (in this case the PVK); the same assumption can be made for LUMO, which should correspond to the LUMO level of OXD-7. With these assumptions, we expected a small hole-blocking barrier at the EML/TmPyPb interface. More complicated was estimation of the electrical carrier mobility. Liu et al. [51] demonstrated a power dependence on the concentration of the individual materials in the matrix and considered that an electrical carrier in the matrix has a mobility that can be given by $\mu_{mix}=\mu_{1}^{C_{1}}\times \mu_{2}^{C_{2}}\times \ldots \times \mu_{m}^{C_{m}}$, where 1, 2, …, *m* corresponds to the different materials and *C* is their individual concentration in the host:guest matrix, where *C*_1_ + *C*_2_ + … + *C_m_* = 1. The expression is valid for both electrons and holes.

It is also known that the effective mobility of an electrical carrier in an organic semiconductor depends critically on the molecular ordering and stacking [52]. Electrical carriers can also be subjected to space-charge conditions, and to the effect of energy levels acting as traps, which primarily tends to decrease mobility [53]. Therefore, allowing a high molecular order stacking is important, particularly when the electron and hole density profiles in the OLED active layer, depending on the mobility, are fundamental for the correct exciton confinement and efficient recombination. For solution-deposited layers, the chemical characteristics of the solutions and the physical characteristics of solvent evaporation rate and temperature (besides the environmental atmospheric conditions) play a fundamental role in allowing the best molecular ordering, critically contributing to a homogeneous order (at the molecular scale) of the final film. Consequently, the efficiency of solution-deposited devices can be improved via the choice of solvent and evaporation temperature, rate, and atmospheric environment. Although particularly complex, the framework can be critically improved.

#### 3.2.1. Devices with an Emissive Layer Using Chloroform as a Solvent

**Figure 4:**
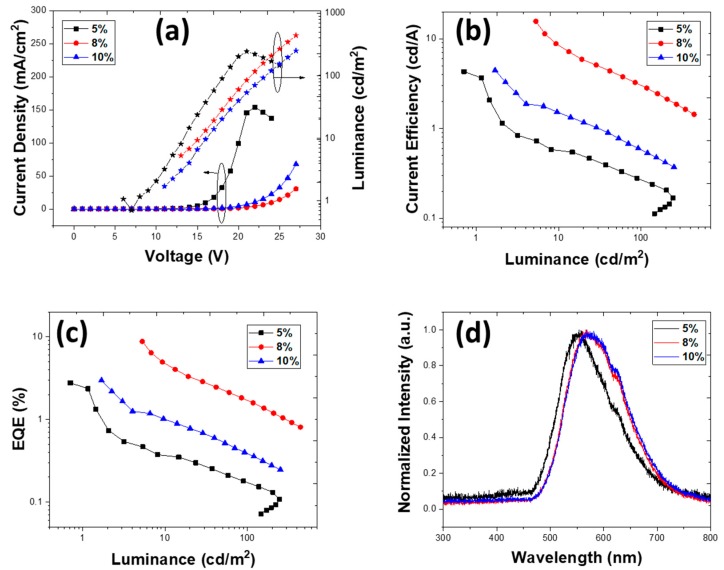
Device characteristics for structure ITO/PEDOT:PSS (40 nm)/PVK:OXD-7:TXO-TPA (x wt.%) (40 nm)/TmPyPb (40 nm)/LiF (1 nm)/Al (100 nm) deposited from chloroform. (**a**) Current density vs. voltage vs. luminance, (**b**) current efficiency vs. luminance, (**c**) EQE vs. luminance, and (**d**) electroluminescence spectra for 5, 8, and 10 wt.% (at 20 V).

The deposition parameters were optimized to maximize the film homogeneity. Despite that, devices fabricated using chloroform as a solvent exhibited high resistivity. In chloroform, the lower viscosity of 0.58 cP and extremely fast evaporation rate of the solvent due to its high volatility led to formation of a non-homogeneous and poorly molecular ordered film of TXO-TPA in a PVK:OXD-7 matrix (Figure 3a) with the highest root-mean-square roughness. Figure 4 shows the results obtained for the device structure ITO/PEDOT:PSS (40 nm)/EML (40 nm)/TmPyPb 40 nm)/LiF (1 nm)/Al (100 nm). The doping concentrations were 5, 8, and 10 wt.%.

The devices with 40 nm ETL and 40 nm EML exhibited a higher turn-on voltage of 13 V, showing the OLEDs’ higher resistivity. Surprisingly, the fabricated OLEDs did not show changed device characteristics upon changing the thickness of the emissive layer. The device with both ETL and EML of 40 nm showed an EQE of 2.77%, 8.78%, and 2.97%, while the device with 25 nm EML exhibited EQE of 2.72%, 7.32%, and 3.37% for 5, 8, and 10 wt.%, respectively (Figure S2, Supplementary Materials).

The Commission Internationale de l’Eclairage (CIE 1931) chromaticity coordinates (*x*, *y*) of OLED were determined to be (0.50, 0.47) for 8 wt.%. In the CF solvent for the device with 40 nm EML, the luminance was relatively low at 400 cd/m^2^, which might have been due to poor molecular ordering of the films, which caused leakage of the current and reduced exciton formation. All the devices exhibited emission at 570 nm, corresponding to emission from TXO-TPA. In addition, emission spectra showed a red-shift observed upon TADF doping concentration increase, which is common [42]. In the device with an EML thickness of 25 nm, the EL spectra changed and red-shifted to 580 nm (Figure S2, Supplementary Materials), and this difference in the EL peaks could have been due to the effect of different electrical charge density profiles in thin and thicker EMLs and the solvatochromism effect [43]. An additional shoulder at 625 nm was also observed in the EL spectra following the TADF emission, as described earlier [25]. In the thick 40 nm EML, it seemed that the recombination zone was better confined in the EML, increasing the radiative relaxation probability and further improving the device performance.

It is worth mentioning that the EQE had a maximum value at >1 cd/m^2^, and the device with 5 wt.% was the lowest at low luminance. Table 2 shows the main results for the OLEDs with different active layer thicknesses deposited using CF as a solvent.

In any case, the results, independently of the very simplified device structure, were poor and did not effectively correspond to the results expected from this TADF emitter (based on the PL data). The main explanation was to the difficulty of achieving an optimized hole and electron density profile in the active layer, suitable for final efficient exciton recombination. The nature of the active layer morphology, due to probably non-ordered molecular stacking, led to an electrically unbalanced device (because of deep changes in electrical carrier mobility from the Pool–Frenkel model [54]). It should be noted that no further optimization was achieved by changing temperature and/or evaporation rate and/or active layer thickness (via spin-coater parameters). This simple conclusion is also supported by the fact that changing ETL thickness did not result in significant differences, supporting the idea that the main issue was related to the EML itself.

#### 3.2.2. Devices with an Emissive Layer Using Dichlorobenzene as a Solvent

**Figure 5:**
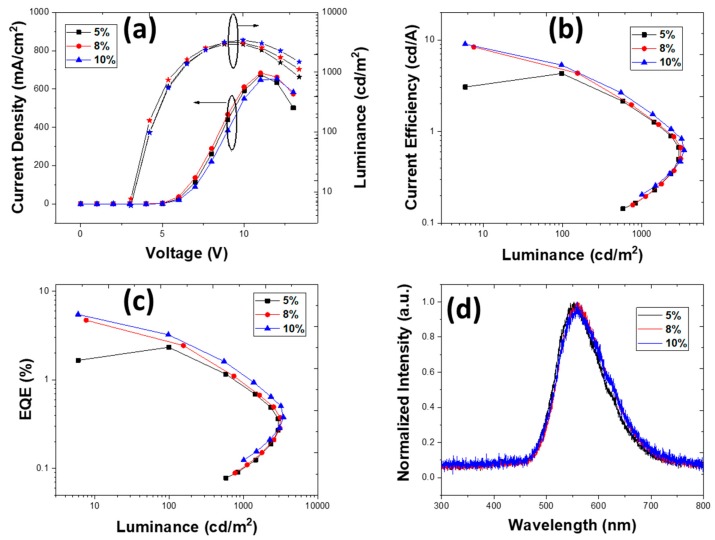
Device characteristics for structure ITO/PEDOT:PSS (40 nm)/PVK:OXD-7:TXO-TPA (x wt.%) (25 nm)/TmPyPb (40 nm)/LiF (1 nm)/Al (100 nm) deposited from dichlorobenzene. (**a**) Current density vs. voltage vs. luminance, (**b**) current efficiency vs. luminance, (**c**) EQE vs. luminance, and (**d**) electroluminescence spectra for 5, 8, and 10 wt.% (at 14 V).

**Figure 6:**
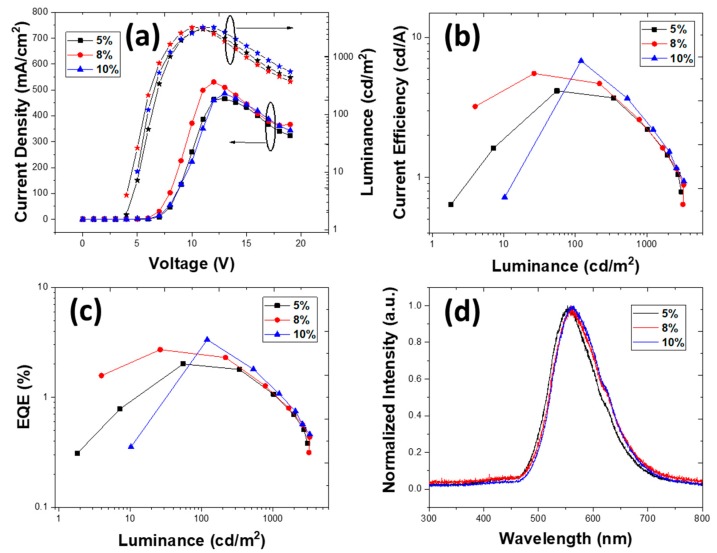
Device characteristics for structure ITO/PEDOT:PSS (40 nm)/PVK:OXD-7:TXO-TPA (x wt.%) (40 nm)/TmPyPb (40 nm)/LiF (1 nm)/Al (100 nm) deposited from dichlorobenzene. (**a**) Current density vs. voltage vs. luminance, (**b**) current efficiency vs. luminance, (**c**) EQE vs. luminance, and (**d**) electroluminescence spectra for 5, 8, and 10 wt.% (at 14 V).

Dichlorobenzene is a more viscous solvent with a higher boiling point than CF. The device configuration with the DCB solvent for EML was ITO/PEDOT:PSS (40 nm)/EML (*X* nm)/TmPyPb (*Y* nm)/LiF (1 nm)/Al (100 nm), where *X* = 25 and 40 and *Y* = 30 and 40 nm. The doping concentrations were 5, 8, and 10 wt.%. We kept the same device structures as were used with CF as a solvent. The viscosity of DCB is 1.32 cP with a higher evaporation rate, which changed the film formation, and was expected to change the electrical and optical properties of the film. The device with both EML and ETL of 40 nm exhibited a lower V_ON_ of 4 V, compared to its chloroform counterpart, but the EQE reduced to 2%, 2.71%, and 3.34% in 5, 8, and 10 wt.% doping, respectively. Figure 5 and Figure 6 show that upon increasing the thickness of EML, the resistivity increased monotonically.

Surprisingly, the characteristics of the OLEDs—EQE, brightness, or turn-on voltage—did not change substantially with EML thickness. When the thickness of EML was reduced to 25 nm, the device EQE was 2.33%, 4.71%, and 5.47% at 5, 8, and 10 wt.%, respectively. In DCB, upon reducing the thickness there was no significant difference in the device performance, and at 10 wt.% TXO-TPA, the EQE increased from 3.34% to 5.47% and the luminance from 3279 cd/m^2^ to 3427 cd/m^2^.

This decrease in the OLED EQE compared to chloroform can be attributed due to the much denser thin film achieved with DCB, where the π–π stacking of carbazoles in PVK was increased compared to the minimized stacking in chloroform [55]. Moreover, the propensity to form aggregates (Figure 3b) with poor film morphology (although the root-mean-square roughness was less than one-half that of the films deposited with CF as a solvent) should lead to an increase of defects in the active layer with further loss of radiative recombination, independently of the less resistive device. Therefore, these devices exhibited reduced EQE performance compared to those that used CF as a solvent for the active layer. Table 3 summarizes the main results depending on the ETL thickness and TXO-TPA concentration.

It is clear that the characteristics achieved were still unsatisfactory. Two aspects should be highlighted in the direct comparison of OLEDs with active layers deposited from solutions with CF or DCB solvents: the turn-on voltage and brightness. Using DCB as a solvent, a remarkable reduction in V_ON_ was achieved. Under space-charge conditions, the transition from an ohmic behavior to an SCLC depends, in the simplest model, on the electrical carrier density and effective electrical mobility (which can depend on energy trap levels). Such a sharp decrease in the V_ON_ value implies a pronounced change in carrier density and mobility, both dependent on film morphology. We still did not have an electrically balanced device (see the low EQE, for instance), but a much better conformation was achieved at the molecular level, allowing higher electrical current densities and better electrical transport. With a correct device structure based on the HOMO/LUMO levels of all organic layers and the work functions of the electrodes, it was clear that the improved electrical carrier transport simultaneously decreased the turn-on voltage, and a high electrical carrier density sharply increased the brightness, although from a microscopic point of view, the morphology (at the film surface) of the EML was not the best.

#### 3.2.3. Devices with an Emissive Layer Using Chlorobenzene as a Solvent

**Figure 7:**
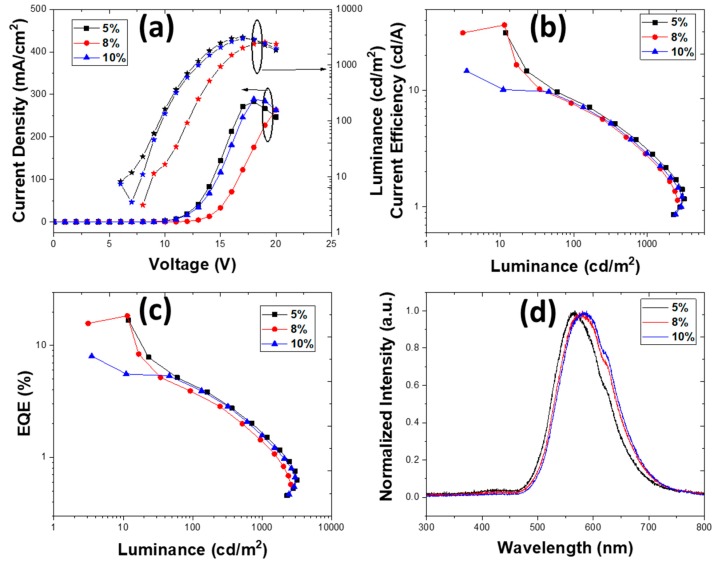
Device characteristics for structure ITO/PEDOT:PSS (40 nm)/PVK:OXD7:TXO-TPA (x wt.%) (40 nm)/TmPyPb (40 nm)/LiF (1 nm)/Al (100 nm) deposited from chlorobenzene. (**a**) current density vs. voltage vs. luminance, (**b**) current efficiency vs. luminance, (**c**) EQE vs. luminance, and (**d**) electroluminescence spectra for 5, 8, and 10 wt.% (at 14 V).

The viscosity of chlorobenzene is 0.80 cP with a moderate evaporation rate, which leads to good thin-film formation and with a lower root-mean-square roughness (Figure 3c). The quantum efficiencies of devices with different solvents were compared under the same dopant conditions. Devices with TmPyPb as the ETL were fabricated with different configurations of both EML and ETL, as previously done with the other solvents. The device configuration was once again ITO/PEDOT:PSS (40 nm)/EML (*X* nm)/TmPyPb (*Y* nm)/LiF (1 nm)/Al (100 nm), where *X* = 25, 40, and 50 nm, and *Y* = 30, 40, and 50 nm. The doping concentrations were 5, 8, and 10 wt.%. The new set of thicknesses appeared after initial observation of overall better performance and a possibility to further improve the key characteristics. The devices with a thickness of 40 nm for both the EML and ETL exhibited excellent device characteristics, with EQE of 16.81%, 18.44%, and 8.03% for 5, 8, and 10 wt.% devices, respectively. All the devices turned on at a lower voltage of 6 V. The EL spectra emitted at 580–585 nm with a shoulder at 623 nm corresponded to a possible emission of the TXO-TPA, as some of our previous simple photoluminescence data showed and as has been confirmed by other authors [25]. An additional, very weak shoulder at 425 nm was also observed, corresponding to the emission of PVK, which is not unusual, and did not influence the electroluminescence profile. A red-shift was also observed on doping concentration, which has been discussed previously [42]. Figure 7 shows the results. The device characteristics are summarized in Table 4.

Upon changing the thickness of the EML, the device performance decreased drastically (Figure S3, Supplementary Materials). When the EML thickness was increased to 50 nm, a lower EQE of 15.33% was observed in the 8 wt.% device, while in the 5 and 10 wt.% devices it was also reduced to 10.6% and 6.14%. No further changes in EL spectra were observed. This reveals that in thicker EMLs, the electrical charge carrier profile led to a more electrically unbalance device, with further non-ideal recombination profiles reducing the radiative recombination probability. When the EML thickness was reduced to 25 nm, the EL spectra exhibited a blue-shift to 565 nm [40,41]. The devices with 5, 8, and 10 wt.% exhibited EQEs of 10.39%, 8.72%, and 7.99%, respectively. All the device characteristics are shown in Figure S4 of the Supplementary Materials.

Along with a change in the thickness of EML, the effect of changed ETL thickness on device performance was also observed. When the thickness of TmPyPb was reduced to 30 nm, the performance reduced to EQEs of 11.65%, 14.53%, and 10.27% in 5, 8, and 10 wt.% TXO-TPA devices. A blue-shift in EL spectra was observed at 575 nm, for which the previous explanation was also valid. The decrease of the EQE can be simply explained by the (again) electrically unbalanced device. We will discuss this later. The results can be viewed in Figure S5 of the Supplementary Materials.

On the other hand, when the ETL thickness was increased to 50 nm, the device performance improved compared to an ETL of 25 nm, although it remained lower than for the device with an ETL of 40 nm. An EQE of 13.73%, 15.82%, and 12.23% was observed for the 5, 8, and 10 wt.% devices, respectively. In the EL spectra, a red-shift was observed at 590 nm. Nevertheless, this configuration exhibited lower EQE compared to the devices both EML and ETL of 40 nm. From the results, we can conclude that with EML and ETL of 40 nm, better electrical carrier injection and transport was observed, which led to the confinement of excitons in the recombination region, resulting in enhanced performance. All the devices turned on lower at 5 V; the main results can be observed in Figure S6 of the Supplementary Materials.

Comparing our best results with the equivalent found in the literature for this TADF [25], we recorded almost the same maximum EQE (around 18%, in our case at slightly higher brightness) and a similar turn-on voltage, showing that our devices had balanced charge injection/transport. We recorded a significantly lower brightness, although in our case we measured the brightness only in the normal emitting direction. Additionally, a simple theoretical estimate of the maximum expected EQE using TXO-TPA can be made, as for practical applications EQE ~$\;\eta_{out}\;\times {\;\eta }_{int}$. The internal efficiency can be approximated by $\eta_{int}=\gamma \times \eta_{ST}\times \Phi_{PL}$, where γ corresponds to the charge-balance factor (we usually assume γ = 1 for electrically balanced devices), ϕ_PL_ is the PLQY (ϕ_PL_ = 0.60 ± 0.33 for the PVK:OXD-7: 8 wt.% TXO-TPA, see Table 1), and η_ST_ is the efficiency of exciton creation (η_ST_ = 1 for TADF-type devices, as usual). As for the outcoupling efficiency η_out_, we considered a conservative value of 0.3. Therefore, the maximum theoretical EQE for our device was nearly 19%, in line with our experimental EQE of 18.4 ± 0.5%. This means that with careful development of the host:guest matrix in order to improve both the PLQY and the charge transport and exciton confinement in EML, we were able to achieve the maximum external efficiency possible, similar to the best values reported in the literature for a more complex device structure.

In order to verify the effectiveness of both exciton confinement in EML and electrical balance transport, an attempt to achieve a more balanced electron density profile across all device layers (hole mobility of all materials is almost low) was made using a different ETL. We needed to ensure that the triplet level was relatively high and the electron mobility was in the range 10^−5^–10^−4^ cm^2^V^−1^s^−1^. TPBi, with its relatively low HOMO level, fulfilled these requirements. The devices were fabricated using this material as an ETL in order to study the direct improvement in the EML when deposited from solution using CB as a solvent. Curiously, these OLEDs exhibited an EQE of 11.95% for 8 wt.%, an impressive result, considering that no hole blocking was expected at the EML/TPBi interface. As referenced, the electron mobility of TPBi is lower than TmPyPb, but a relatively electrically balanced device was apparently achieved. Nevertheless, the practical absence of the hole-blocking barrier at the EML/ETL interface seemed to be a drawback, indicating that, as our previous work has shown, TADFs appear to be able to modulate the electrical carrier mobility in a host:guest EML in a strong way.

**Figure 8:**
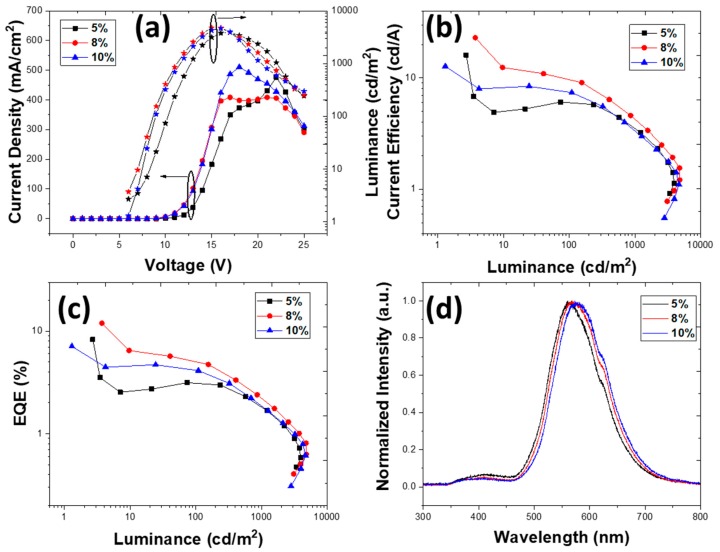
Device characteristics for structure ITO/PEDOT:PSS (40 nm)/PVK:OXD7:TXO-TPA (x wt.%) (40 nm)/TPBi (40 nm)/LiF (1 nm)/Al (100 nm) deposited from chlorobenzene. (**a**) Current density vs. voltage vs. luminance, (**b**) current efficiency vs. luminance, (**c**) EQE vs. luminance, and (**d**) electroluminescence spectra for 5, 8, and 10 wt.% (at 14 V).

The devices with TPBi as ETL exhibited an L_max_ of 4809 cd/m^2^ in a device structure that followed the ones previously used in this work: ITO/PEDOT:PSS (40 nm)/EML (40 nm)/TPBi (40 nm)/LiF (1 nm)/Al (100 nm). The device turned on at 5.5 V. The devices with 5 wt.% and 10 wt.% exhibited a lower performance, with EQEs of 8.3% and 3.34%, respectively. The electroluminescence spectra peaked at 563, 570, and 580 nm for the 5, 8, and 10 wt.% devices, respectively, while a red-shift was observed in the EL spectra with an increase of TXO-TPA doping concentration. The color coordinates of the devices with 5, 8, and 10 wt.% were very similar. The results are shown in Figure 8 and summarized in Table 5.

Although we achieved interesting results (the best results being obtained with 8 wt.% TADF, according to our previous experiments), we were relatively far from the results obtained with TmPyPb as an ETL. A concise explanation can be given if we consider that, independently of a balanced electrical carrier’s mobility, the confinement in the emissive layer forced by interface carrier blocking is the best way to improve the OLED key characteristics. Simultaneously, this could help to further design efficient and simple device architectures based on solution-deposition methods. From the results, it is clear that the best devices were obtained using CB as a solvent (for 8 wt.% TXO-TPA in the active layer) and with EML/ETL thicknesses of 40 nm/40 nm. The effect of the hole blocking at EML/TmPyPb interface seemed to be a clear advantage, allowing better exciton confinement in EML.

Considering our best device structure, we were able to draw some conclusions. It is widely known that an optimized OLED structure should allow (besides good electrical charge injection) efficient electrical charge transport and further exciton confinement in the active layer. The increase in efficiency directly depends on the ratio of emitted photons to injected electrical carriers. In the devices, both anode (ITO/PEDOT:PSS) and cathode (LiF/Al) were optimized to increase the electrical charge injection. In addition, the electrical transport must be optimized. In general, the electrical properties of the different organic semiconductors determined both electron and hole density profile across the devices. These profiles should guarantee a high density of exciton and confinement in a symmetrical recombination profile in the active layer. These assumptions will ensure the highest possible probability of the radiative recombination. In this framework, electrical charge mobility and organic layer thickness play a fundamental role. The desirable condition is that both electron and hole mobility should be (under the same electrical field conditions, following the Poole–Frankel model) as similar as possible. In practice, and because these conditions are sometimes far from ideal, it is essential to optimize the thickness of the organic layers to guarantee that the abovementioned conditions are achieved. This simple approach was followed in this work. Although important, as the results showed, further improvement can be made. Firstly, including both p- and n-type host materials in the active layer, we were able modulate the electron and hole mobility straightforwardly (following the previously mentioned conditions for the electrical mobilities of an organic layer composed of a mixture of materials). Secondly, electrical transport in organic materials is strongly dependent on the molecular ordering and conformation, which can be improved using various solvents to alter the molecular stacking. In our case, the conjugation of these concepts led to excellent results, if we consider that the device structure was very simple. Naturally, we took into account the direct influence of energy levels from defects acting as traps. From the *J*–*V* data, it seems that all devices should have deep traps (as usual), and we assume that their influence will decrease as the molecular stacking improves.

Comparable results have only been achieved to date in more complex structures using a thermal evaporation process, which attempts to compensate the natural absence of a balanced electrical carrier’s mobility, with an increase of organic layers in order to obtain the final desired exciton profile density inside the active layer.

## 4. Conclusions

In summary, for efficient generation of solution-processed OLEDs based on a TXO-TPA emitter, and in a very simplified device structure of only two organic layers, we studied the solvents chlorobenzene, dichlorobenzene, and chloroform in generation of a film with better molecular ordering in order to enhance the electrical properties of the emissive layer. Using the electron/hole mobility properties of all organic materials to construct an efficient device, together with organic film thickness adjustment, we were able to change the expected electron/hole density profile across the devices, particularly in the emissive layer. The optimized doping concentration of emitter and the thickness of both EML and ETL provided information on recombination region formation and predicted EML profiles. This led to a high EQE of 18.44% and an η_c_ of 36.71 cd/A, in line with the best results found for this specific TADF emitter using the thermal evaporation process in a much more complex device structure. An insight into the photophysical results was provided by comparison of the theoretical EQE values with the experimental values. With these results, we believe that the presented approach provides a new and exciting framework for further enhancements in these particular highly efficient emitters, with potential applications in both efficient lighting and display applications, with a noticeable reduction of fabrication costs and process.

## Figures and Tables

**Table 1:** Photoluminescence quantum yield (PLQY) of films (host PVK:OXD-7) in air and oxygen-free conditions.

PLQY (±3%)	1% TXO-TPA	2% TXO-TPA	5% TXO-TPA	8% TXOTPA	10% TXO-TPA
PLQY (in air) %	10	12	14	18	15
PLQY (oxygen free) %	18	23	52	60	54

**Table 2:** Summary of results obtained for device with structure ITO/PEDOT:PSS (40 nm)/PVK:OXD-7:TXO-TPA (x wt.%) (X nm)/TmPyPb (40 nm)/LiF (1 nm)/Al (100 nm) where X = 25 or 40, deposited with chloroform. (Max EQE values at > 5 cd/m^2^).

TXO-TPA Wt.%	η_c_ (cd/A)	η_p_ (Lm/W)	EQE (%)	L_max_ (cd/m^2^)	*J_ON_* (mA/cm^2^)	V_ON_ (V)	CIE (x,y)
**EML thickness of 40 nm**							
5	4.31	3.04	2.77	245	1.64 × 10^−2^	14	0.43, 0.51
8	15.66	3.78	8.78	440	3.37 × 10^−2^	13	0.50, 0.47
10	4.46	1.27	2.97	416	3.38 × 10^−2^	11	0.50, 0.47
**EML thickness of 25 nm**							
5	4.86	1.17	2.72	353	1.64 × 10^−2^	13	0.43, 0.51
8	11.39	2.38	7.32	225	3.37 × 10^−2^	13	0.50, 0.47
10	5.06	1.32	3.37	472	3.38 × 10^−2^	12	0.50, 0.47

**Table 3:** Summary of results obtained for device with structure ITO/PEDOT:PSS (40 nm)/PVK:OXD-7:TXO-TPA (x wt.%) (X nm)/TmPyPb (40 nm)/LiF (1 nm)/Al (100 nm) where X = 25 or 40, deposited from dichlorobenzene. (Max EQE values at > 10 cd/m^2^).

TXO-TPA Wt.%	η_c_ (cd/A)	η_p_ (Lm/W)	EQE (%)	L_max_ (cd/m^2^)	*J_ON_* (mA/cm^2^)	V_ON_ (V)	CIE (x,y)
**EML thickness of 40 nm**							
5	4.15	2.17	2.00	3038	1.33	4	0.40, 0.53
8	5.52	3.46	2.71	3268	4.70 × 10^−1^	4	0.43, 0.52
10	6.80	3.52	3.34	3279	1.76	4.5	0.44, 0.52
**EML thickness of 25 nm**							
5	4.29	2.70	2.33	2939	2.28	4	0.40, 0.50
8	8.36	6.57	4.71	3115	9.0 × 10^−2^	4	0.40, 0.48
10	9.00	7.07	5.47	3427	6.57 × 10^−2^	4	0.40, 0.46

**Table 4:** Summary of results obtained for device with structure ITO/PEDOT:PSS (40 nm)/PVK:OXD7:TXO-TPA (x wt.%) (A nm)/TmPyPb (B nm)/LiF (1 nm)/Al (100 nm) deposited from chlorobenzene. (Max EQE values at >5 cd/m^2^) (A = 25, 40, 50 nm, B = 30, 40, 50 nm).

Wt.% *A = EML B = ETL*	η_c_ (cd/A)	η_p_ (Lm/W)	EQE (%)	L_max_ (cd/m^2^)	*J_ON_* (mA/cm^2^)	V_ON_ (V)	CIE (x,y)
**A = 25 nm, B = 40 nm**							
5	21.00	9.42	10.39	3237	1.96 × 10^−2^	6	0.44, 0.51
8	17.21	9.01	8.72	3878	2.53 × 10^−2^	6	0.46, 0.50
10	10.95	7.95	7.99	3123	4.33 × 10^−2^	6	0.47, 0.49
**A = 40 nm, B = 40 nm**							
5	31.60	14.18	16.81	3180	3.75 × 10^−2^	6	0.45, 0.51
8	36.71	12.81	18.44	2585	3.11× 10^−2^	6	0.48, 0.49
10	14.74	6.61	8.03	2992	2.37× 10^−2^	6	0.49, 0.48
**A = 50 nm, B = 50 nm**							
5	19.22	8.62	10.60	2240	1.90 × 10^−2^	6	0.46, 0.50
8	29.24	13.12	15.33	1700	4.33 ×10^−3^	6	0.49, 0.48
10	6.14	4.36	6.14	2309	1.05 × 10^−1^	6	0.49, 0.48
**A = 40 nm, B = 30 nm**							
5	22.41	11.73	11.65	2885	8.90 × 10^−3^	6	0.44, 0.51
8	29.47	13.22	14.53	2513	5.57 × 10^−3^	6	0.47, 0.50
10	19.34	8.68	10.27	3021	2.11 × 10^−2^	6	0.48, 0.49
**A = 40 nm, B = 50 nm**							
5	26.21	11.76	13.73	1490	8.0 × 10^−3^	6	0.46, 0.50
8	28.60	14.97	15.82	3800	1.95 × 10^−2^	6	0.49, 0.48
10	22.50	8.83	12.23	1922	2.04 × 10^−2^	7	0.48, 0.49

**Table 5:** Summary of results obtained for device with structure ITO/PEDOT:PSS (40 nm)/PVK:OXD7:TXO-TPA (x wt.%) (40 nm)/TPBi (40 nm)/LiF (1 nm)/Al (100 nm) deposited from chlorobenzene. (Max EQE values at > 5 cd/m^2^).

TXO-TPA Wt.%	η_c_ (cd/A)	η_p_ (Lm/W)	EQE (%)	L_max_ (cd/m^2^)	*J_ON_* (mA/cm^2^)	V_ON_ (V)	CIE (x,y)
5	16.07	8.41	8.3	3950	1.67 × 10^−2^	5.5	0.43, 0.50
8	22.97	12.02	11.95	4809	1.61 × 10^−2^	5.5	0.46, 0.50
10	12.27	6.66	7.19	4681	1.02 × 10^−2^	5.5	0.46, 0.51

## Supplementary Materials

The following are available online at https://www.mdpi.com/2079-4991/10/1/101/s1, Figure S1: AFM Image of PVK:OXD-7:TXO-TPA of wt.%; (a) 5%, (b) 8%, and (c) 10% in dichlorobenzene, and (d) 5%, (e) 8%, and (f) 10% in chlorobenzene, Figure S2: Device characteristics for structure ITO/PEDOT:PSS (40 nm)/PVK:OXD7:TXO-TPA (x wt.%) (25 nm)/TmPyPb (40 nm)/LiF (1 nm)/Al (100 nm) deposited from chloroform. (a) current density vs. voltage vs. luminance, (b) Current efficiency vs. luminance, (c) EQE vs. luminance and (d) electroluminescence spectra for 5,8 and 10 wt.% (at 20 V), Figure S3: Device characteristics for structure ITO/PEDOT:PSS (40 nm)/PVK:OXD7:TXO-TPA (x wt.%) (50 nm)/TmPyPb (40 nm)/LiF (1 nm)/Al (100 nm) deposited from chlorobenzene. (a) current density vs. voltage vs. luminance, (b) Current efficiency vs. luminance, (c) EQE vs. luminance and (d) electroluminescence spectra for 5, 8 and 10 wt.% (at 20 V), Figure S4: Device characteristics for structure ITO/PEDOT:PSS (40 nm)/PVK:OXD7:TXO-TPA (x wt.%) (25 nm)/TmPyPb (40 nm)/LiF (1 nm)/Al (100 nm) deposited from chlorobenzene. (a) current density vs. voltage vs. luminance, (b) Current efficiency vs. luminance, (c) EQE vs. luminance and (d) electroluminescence spectra for 5, 8 and 10 wt.% (at 20 V), Figure S5: Device characteristics for structure ITO/PEDOT:PSS (40 nm)/PVK:OXD7:TXO-TPA (x wt.%) (40 nm)/TmPyPb (30 nm)/LiF (1 nm)/Al (100 nm) deposited from chlorobenzene. (a) current density vs. voltage vs. luminance, (b) Current efficiency vs. luminance, (c) EQE vs. luminance and (d) electroluminescence spectra for 5, 8 and 10 wt.% (at 20 V) and Figure S6: Device characteristics for structure ITO/PEDOT:PSS (40 nm)/PVK:OXD7:TXO-TPA (x wt.%) (40 nm)/TmPyPb (50 nm)/LiF (1 nm)/Al (100 nm) deposited from chlorobenzene. (a) current density vs. voltage vs. luminance, (b) Current efficiency vs. luminance, (c) EQE vs. luminance and (d) electroluminescence spectra for 5, 8 and 10 wt.% (at 20 V).
