# Supplementary material for: Towards Highly Efficient TADF Yellow-Red OLEDs Fabricated by Solution Deposition Methods: Critical Influence of the Active Layer Morphology
Source: Nanomaterials (Basel). 2020 Jan 4;10(1):101. doi: 10.3390/nano10010101 (PMC7022318; doi:10.3390/nano10010101)
Supplement: Supplementary file 1 [file nanomaterials-10-00101-s001.pdf]

nanomaterials

Supplementary Information

Towards high efficient TADF yellow-red OLEDs fabricated by solution deposition methods: critical influence of the active layer morphology

Manish Kumar [1,2] and Luiz Pereira [1,*]

[1] Nanomodulation and Nanofabrication, Department of Physics and i3N—Institute for Nanostructures, University of Aveiro, 3810-193 Aveiro, Portugal; mkumar@ua.pt

[2] CeNTI—Centre for Nanotechnologies and Smart Materials, R. Fernando Mesquita, 2785, 4760-034 Vila Nova de Famalicão, Portugal

* Correspondence: luiz@ua.pt

Received: 22 November 2019; Accepted: 2 January 2020; Published: 4 January 2020

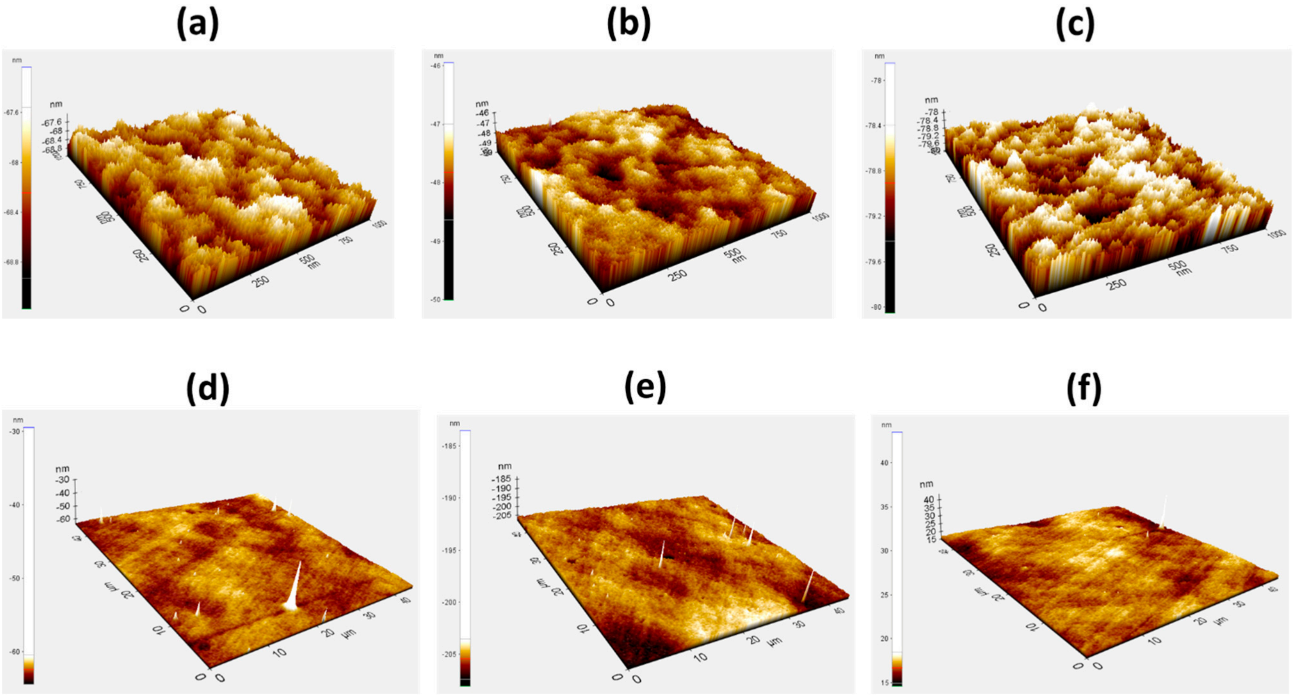

Figure S1. AFM Image of PVK:OXD-7:TXO-TPA of wt.%; (**a**) 5%, (**b**) 8%, and (**c**) 10% in dichlorobenzene, and (**d**) 5%, (**e**) 8%, and (**f**) 10% in chlorobenzene.

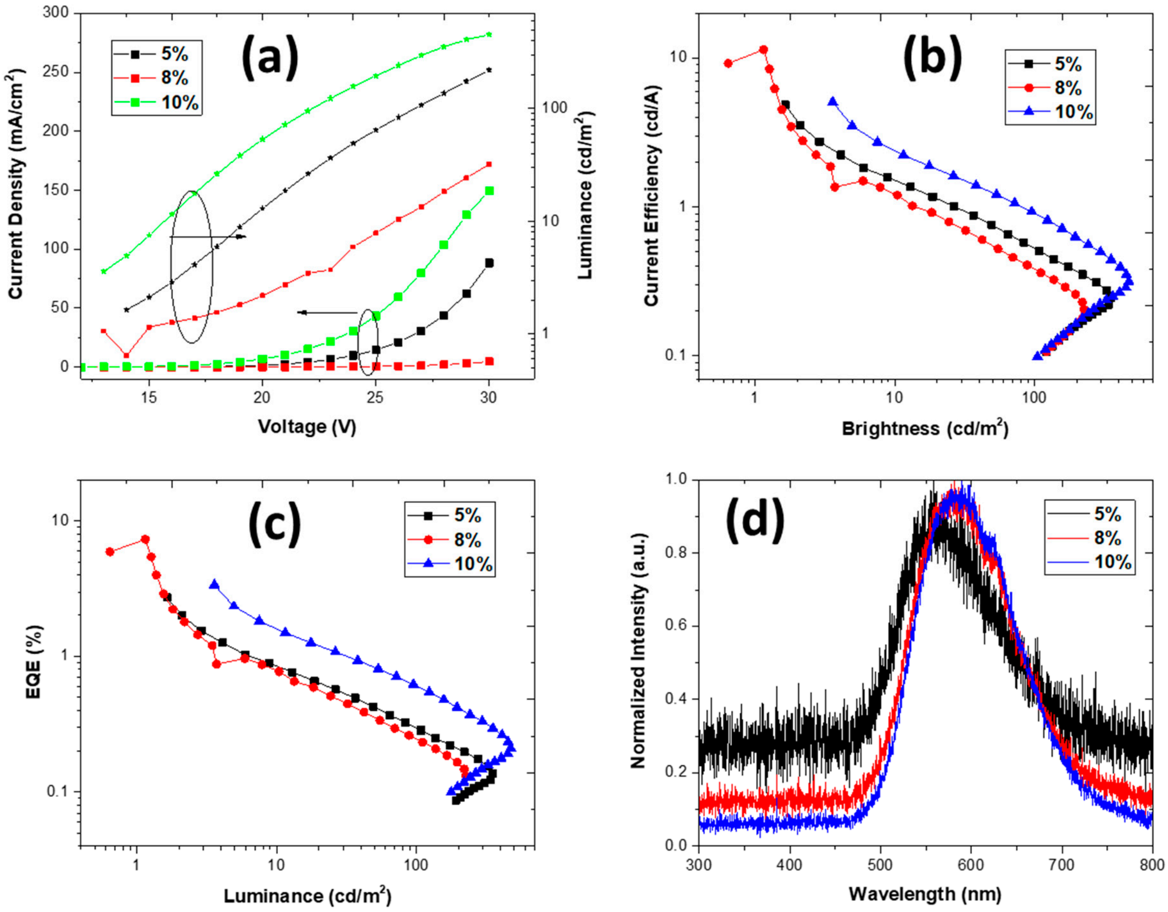

Figure S2. Device characteristics for structure ITO/PEDOT:PSS (40 nm)/PVK:OXD7:TXO-TPA (x wt.%) (25 nm)/TmPyPb (40 nm)/LiF (1 nm)/Al (100 nm) deposited from chloroform. (**a**) current density vs voltage vs luminance, (**b**) Current efficiency vs luminance, (**c**) EQE vs luminance and (**d**) electroluminescence spectra for 5, 8 and 10 wt.% (at 20 V).

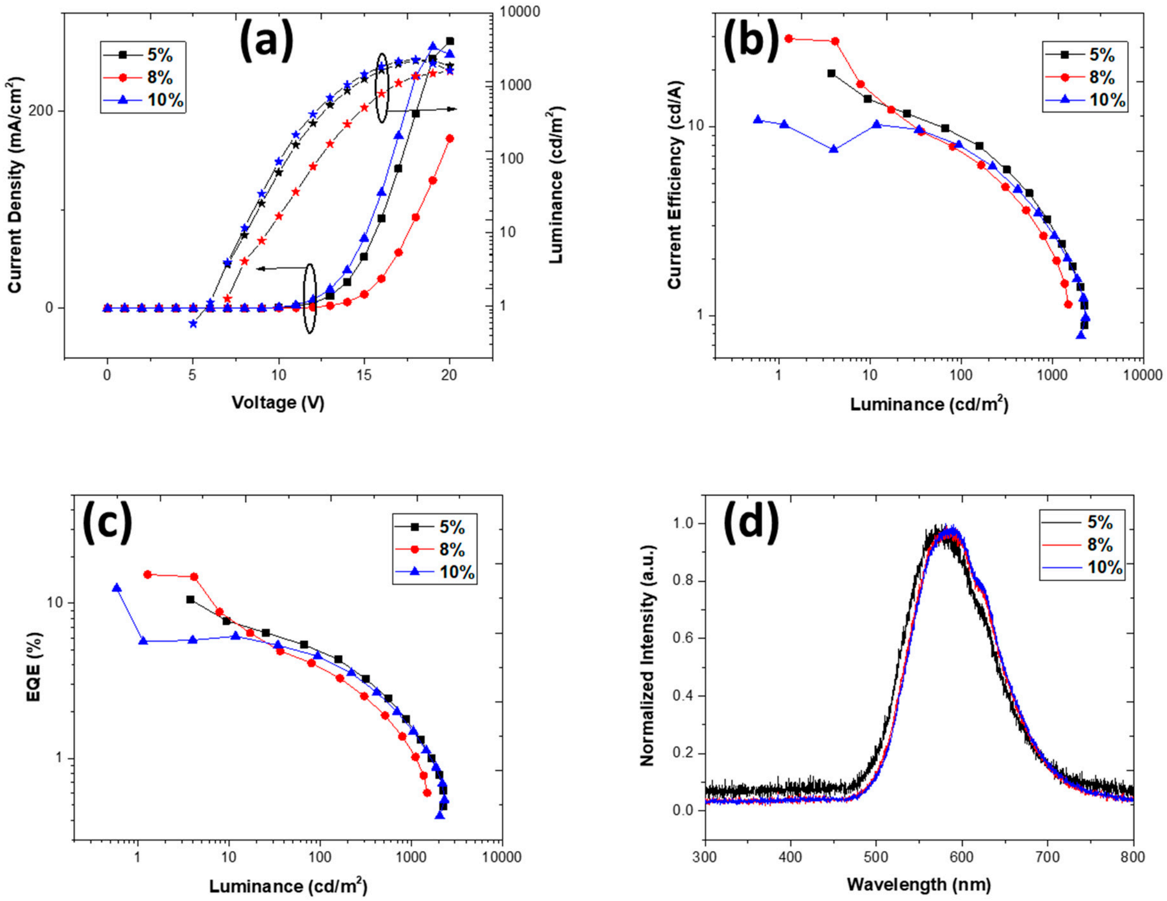

Figure S3. Device characteristics for structure ITO/PEDOT:PSS (40 nm)/PVK:OXD7:TXO-TPA (x wt.%) (50 nm)/TmPyPb (40 nm)/LiF (1 nm)/Al (100 nm) deposited from chlorobenzene. (**a**) current density vs voltage vs luminance, (**b**) Current efficiency vs luminance, (**c**) EQE vs luminance and (**d**) electroluminescence spectra for 5, 8 and 10 wt.% (at 20 V).

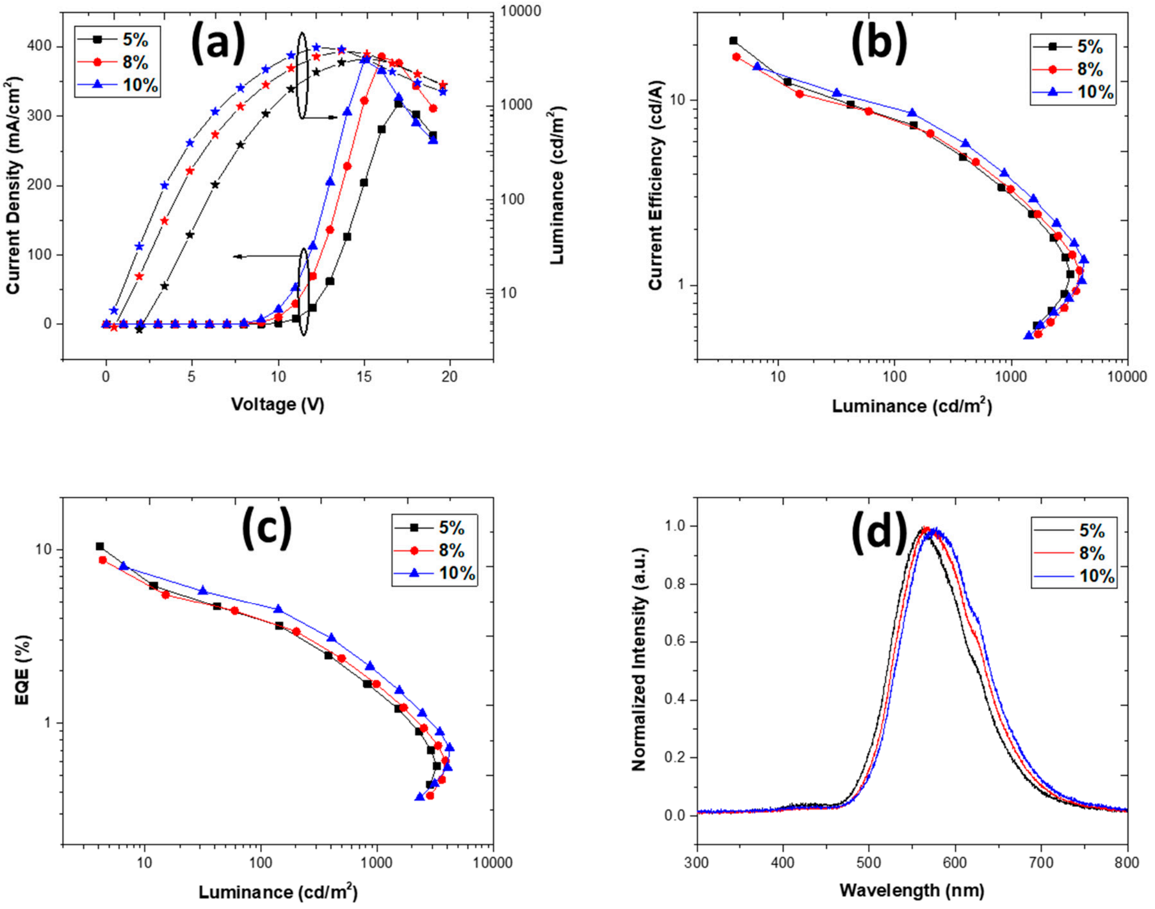

Figure S4. Device characteristics for structure ITO/PEDOT:PSS (40 nm)/PVK:OXD7:TXO-TPA (x wt.%) (25 nm)/TmPyPb (40 nm)/LiF (1 nm)/Al (100 nm) deposited from chlorobenzene. (**a**) current density vs voltage vs luminance, (**b**) Current efficiency vs luminance, (**c**) EQE vs luminance and (**d**) electroluminescence spectra for 5, 8 and 10 wt.% (at 20 V).

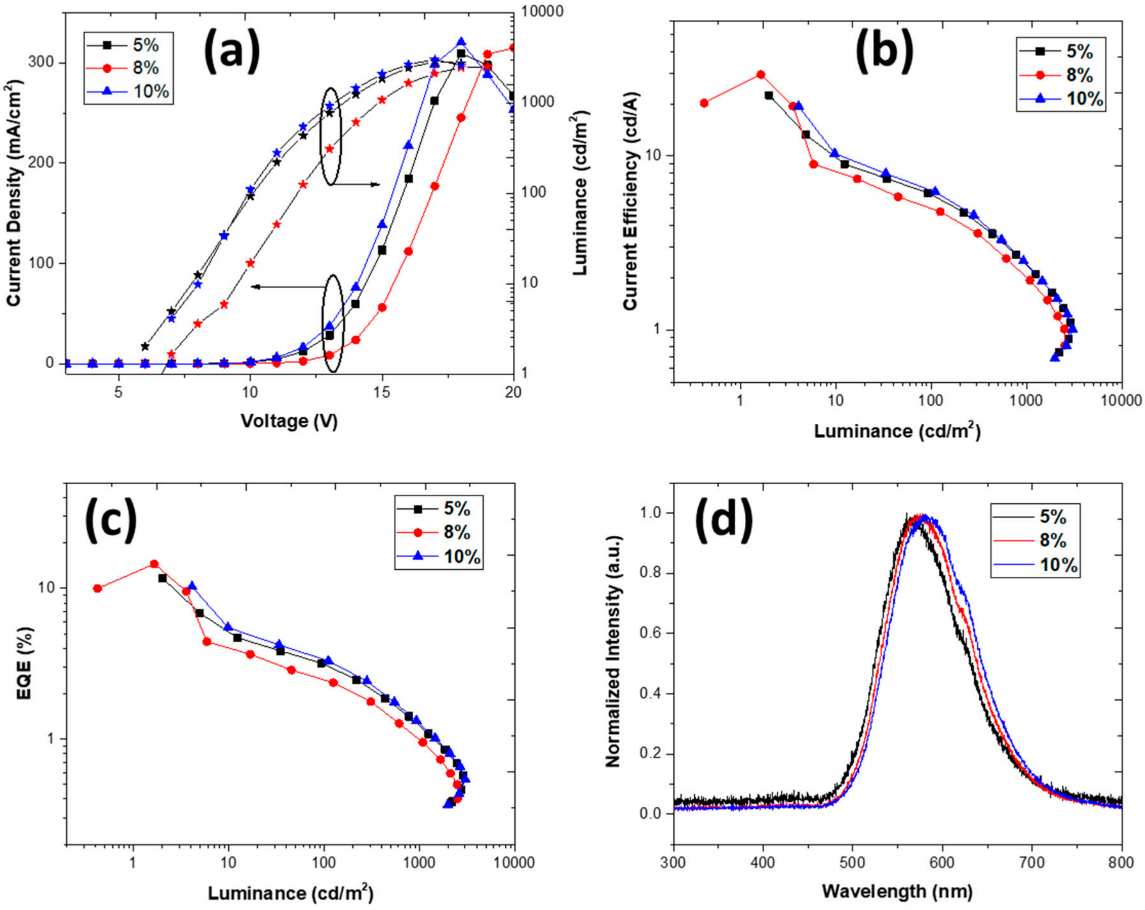

Figure S5. Device characteristics for structure ITO/PEDOT:PSS (40 nm)/PVK:OXD7:TXO-TPA (x wt.%) (40 nm)/TmPyPb (30 nm)/LiF (1 nm)/Al (100 nm) deposited from chlorobenzene. (**a**) current density vs voltage vs luminance, (**b**) Current efficiency vs luminance, (**c**) EQE vs luminance and (**d**) electroluminescence spectra for 5, 8 and 10 wt.% (at 20 V).

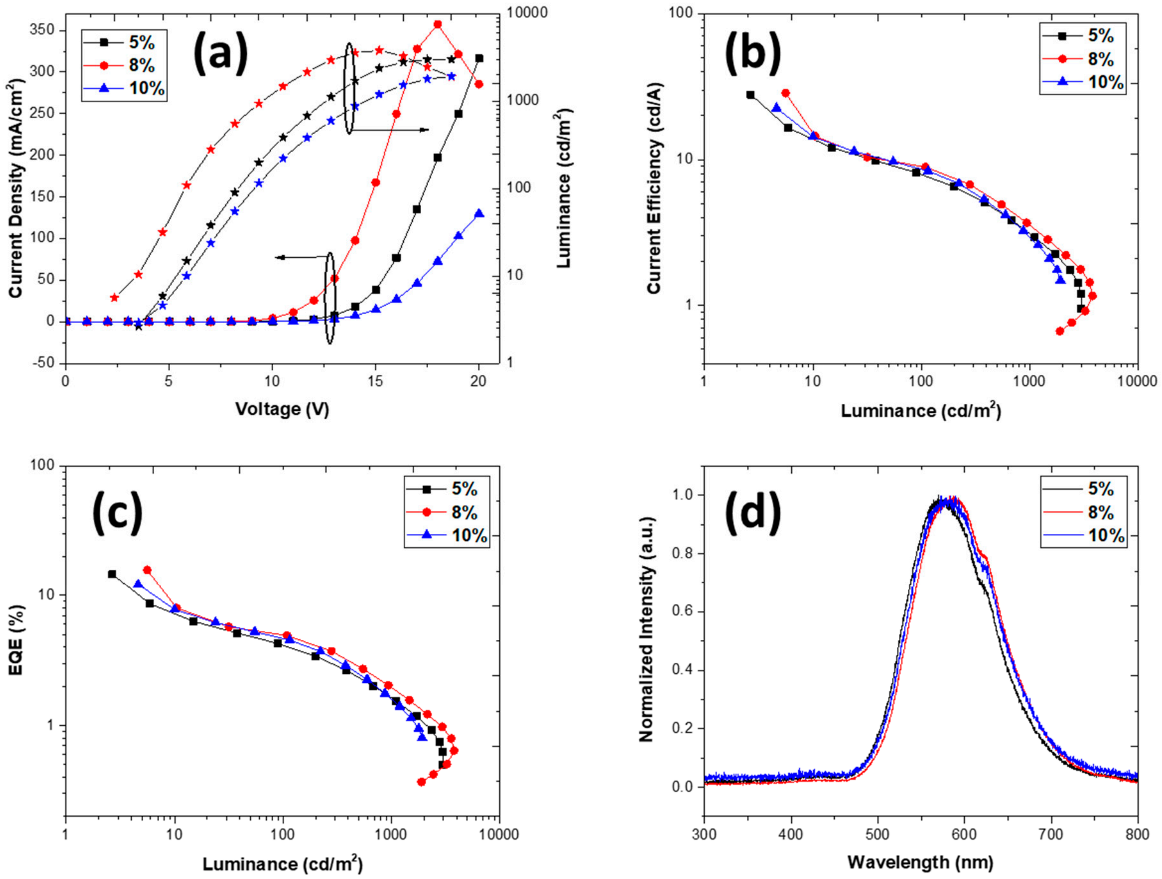

Figure S6. Device characteristics for structure ITO/PEDOT:PSS (40 nm)/PVK:OXD7:TXO-TPA (x wt.%) (40 nm)/TmPyPb (50 nm)/LiF (1 nm)/Al (100 nm) deposited from chlorobenzene. (**a**) current density vs voltage vs luminance, (**b**) Current efficiency vs luminance, (**c**) EQE vs luminance and (**d**) electroluminescence spectra for 5, 8 and 10 wt.% (at 20 V).
